# Herbst Fillings

**Published:** 1887-01

**Authors:** J. P. Geran

**Affiliations:** Brooklyn, N. Y.


### HERBST FILLINGS.

BY DR. J. P. GERAN, BROOKLYN, N. Y.

Read before the Second District Dental Society of the State of New York, November 2, 1886.

Most cheerfully and gratefully do I pay my tribute to the man whose early foresight, skill and inventive genius has worked out and developed a method in dental practice which economizes time, makes labor and work pleasant, and, in a measure, does away with the nervous strain incident to both patient and dentist. Although yet in its infancy, I am convinced that its subsequent advancement will bring forth maturing fruits, by which I trust we all will be benefited.

Filled with many misgivings, with doubts and fears as to the character of his reception, our friend came to our shores to demonstrate a system which had absorbed his time and faculties, and called forth all his latent power and genius. A steadfast friend, unfaltering in his devotion, urged Dr. Herbst to visit America, knowing full well that the warm sympathy of his transatlantic brethren would be extended to him. To-day all his doubts and fears have been dissolved, and whether by the side of the dental chair as demonstrator, or at the festive board, the hand of cordial friendship has been extended and a grateful appreciation accorded him. We are compelled to recognize the fact that the very best years of the doctor's life have been devoted to the fulfillment of his dream, and though all may not agree concerning its merits, and many may be disposed to criticise, as it is their right to do, yet we must be compelled to admit the honesty of purpose and the sincerity and singleness of mind of the man.

If his method be fully investigated and discussed, if prejudice be laid aside in the investigation, there are no fears for its ultimate recognition by the profession. Public opinion must frequently be educated and advanced with a large measure of faith. I am of those who believe in laboring for ultimate good.

New theories, new methods, like fresh seed put in the earth, will, if tested and appreciated, bring forth a harvest; and I congratulate one and all who have given hand and voice to the promotion and advancement of this system.

For many years the dental profession was wedded to hand-pressure for filling teeth with gold, tin, etc., and when the mallet was introduced many condemned it and advanced strong arguments against it, such as the liability to fracture frail and thin walls, the production of inflammation of the periosteum, with the subsequent death of the pulp, and numerous other ills unnecessary to mention. But the thoughtful and progressive men of the age saw the utility of it and urged it forward, and to-day nine-tenths of our operators have adopted the mallet system.

There is no department of science and labor in which there is greater need of scientific and practical training than in dentistry. Success is dependent upon skill, and while a considerable knowledge of general science is not absolutely essential to a man who simply fills teeth, it is a valuable aid which sometimes leads men to avoid blunders costly to their patients and themselves.

To afford an intelligent and satisfactory explanation of the Herbst method would require a far more extended personal experience than it has been my fortune to have. That there is in it something good and practicable, which American dental ingenuity will aid in developing and bringing to perfection, there can be no doubt. My patients are gratified, and most positively declare themselves much pleased with this new method.

In one of the dental journals of last year, Dr. Bödecker said that it would be well to fill about seven-eighths of the cavity by the Herbst method, and finish the masticating surface with the mallet, fearing, I suppose, that the operator would not succeed in making the gold cohere sufficiently to complete the filling with a smooth surface. In a recent conversation with him, he informed me that he now completes the whole operation by the Herbst method, and the experience I have had with it teaches me that the whole filling can be readily done with the rotary instrument. I sometimes make retaining pits, fill them as usual by hand-pressure or the mallet, then add two or three large cylinders, cover them with cotton, and force the whole down or against the cervical wall, moving the instrument freely about the cavities. The retaining pits and the cotton prevent the gold from moving. I then use a smaller rotating instrument for forcing the gold against the sides and the matrix, if one is used. After the bottom is well covered and condensed, I add cylinder after cylinder, burnishing each one down thoroughly before adding another, examining frequently for soft spots, and if any are found, condensing and filling them even with the other part of the filling, keeping all as level as possible, and when the cavities are nearly or quite full, I finish with No. 30 gold.

In contouring the superior laterals, I make the restoration without a matrix. I first cut the usual groove, packing the gold in the undercuts with the mallet, then continuing with the rotating instrument until the cavity is flush with the margin. Then, with a bloodstone rotating point, I burnish on ribbons of Williams' electric gold, making the restoration complete, and the finished filling as dense and hard as if made with either the hand or electric mallet. Unless large pieces are employed, it does not require as much pressure as Dr. Herbst appeared to use. All that is necessary is to anneal the gold slightly, bring each pellet in contact with that which has already been condensed, pass the bloodstone or agate instrument over them a few times, keeping the angles down and the whole as level as possible, and it will be found that a filling has been made quicker than by the old method, and in a manner that will allow finishing with a perfect surface.

